# Targeting fibroblast activation protein in rheumatoid arthritis: from molecular imaging to precision therapeutics

**DOI:** 10.3389/fimmu.2025.1616618

**Published:** 2025-06-18

**Authors:** Yupeng Huang, Yang Wu, Huan Liu, Yuehong Chen, Qibing Xie, Geng Yin

**Affiliations:** ^1^ Department of General Practice, General Practice Medical Center, West China Hospital, Sichuan University, Chengdu, Sichuan, China; ^2^ Department of Rheumatology and Immunology, West China Hospital, Sichuan University, Chengdu, Sichuan, China

**Keywords:** rheumatoid arthritis, fibroblast activation protein, FAPI, disease evaluation, treatment

## Abstract

Rheumatoid arthritis (RA), a chronic inflammatory disorder characterized by synovitis and joint destruction, remains a global health challenge. Activated fibroblast-like synoviocytes (FLS), which play a crucial role in the progression of RA, demonstrate tumor-like invasiveness and secrete inflammatory mediators. Fibroblast activation protein (FAP), a type II transmembrane serine protease, has been extensively studied in oncology for decades and has yielded significant clinical benefits. FAP is highly expressed in tumor-associated fibroblasts and plays a pivotal role in tumor growth, dissemination, and immune escape. In cancer imaging, small-molecule FAP inhibitor (FAPI) PET/CT has demonstrated superior sensitivity for detecting primary tumors and metastases. Additionally, FAP-targeted radionuclide therapy has emerged as a promising strategy for delivering precise radiation to tumors, while sparing healthy tissues. Beyond oncology, research on FAP in non-malignant diseases is rapidly advancing. In RA, FAP is overexpressed in RA-FLS but scarce in normal tissues. Thus, FAPI PET/CT can accurately visualize synovitis and monitor the treatment response in patients with RA. Similarly, FAP imaging is used to assess extra-articular manifestations, such as interstitial lung disease and cardiac fibrosis, by mapping fibroblast activity, offering a non-invasive tool. Moreover, emerging therapies, such as FAP-targeted photodynamic therapy, selectively eliminate pathogenic cells in RA models, highlighting their therapeutic potential. This review highlights the advances in FAP-targeted imaging for RA, specifically focusing on FAP as a key biomarker for diagnosis, disease evaluation, and potential therapeutic use in RA.

## Introduction

Rheumatoid arthritis (RA) is a common chronic inflammatory disease mainly characterized by symmetrical arthritis. Pathological changes include synovitis and vascular opacities, which can cause erosive damage to the bones and joints, ultimately leading to joint deformities. RA may also manifest with extra-articular features such as rheumatoid nodules and vasculitis, potentially involving multiple organ systems, including the cardiovascular, respiratory, neurological, and hematological systems. Serum autoantibodies such as rheumatoid factor (RF) and anti-citrullinated protein antibody (ACPA) are frequently detected in patients with RA. Additionally, RA affects approximately 0.5%–1% of the global population, with a notably higher incidence in women than in men (1, 2), posing a significant risk of disability and imposing a heavy burden on individuals and society.

Currently, the etiology of RA is not fully understood, including genetic predisposition, environmental triggers, and immune system dysregulation. Increasing evidence suggests that fibroblast-like synoviocytes (FLSs) play a crucial role in RA synovitis and joint destruction (3). Under inflammatory stimulation, normal FLSs in the synovium undergo transformation into a pathogenic phenotype, RA-FLS, displaying tumor-like properties such as contact inhibition, resistance to apoptosis, high proliferation rates, and invasion ability. In addition, RA-FLS can secrete large amounts of inflammatory factors and proteases that are directly involved in tissue destruction. These features promote the formation of synovial vascular opacities, persistent synovitis, and joint destruction (4, 5). Based on the pertinent role of FLS in RA joint inflammation and tissue destruction, consuming or clearing activated RA-FLS would be an optimal approach for RA treatment, thereby minimizing adverse reactions associated with immunosuppression. Moreover, this would provide new treatment options for refractory patients whose condition cannot be controlled by existing therapies.

Fibroblast activation protein (FAP) is a cell surface marker of activated RA-FLS, with minimal expression in normal fibroblasts (6–9). Therefore, FAP provides clues regarding the intracellular regulatory mechanisms in RA, and opens up new perspectives for molecular imaging and targeted therapy. Consequently, in this review, we aimed to discuss the potential of targeting FAP to diagnose RA, assess RA disease activity, and evaluate its therapeutic effects on RA.

## Biological functions of fibroblast activation protein

FAP is a 97-kDa type-II transmembrane serine protease expressed on the surface of fibroblasts associated with various diseases and was first discovered by Rettig et al. in 1986 in lung fibroblasts cultured *in vitro* (10). Structurally, FAP consists of 760 amino acid residues, including intracellular, transmembrane, and extracellular domains. The α 2-antiplamin cleansing enzyme (APCE) is produced by post-translational cleavage and is the extracellular part of FAP (11). As a member of the propyl peptidase family, which contains FAP, dipeptidyl peptidase IV (DPPIV), DPP7, DPP8, DPP9, and prolyl carboxypeptidase (PCP), FAP shares 70% amino acid sequence homology with DPPIV (12). FAP also exhibits dipeptidyl peptidase and endopeptidase activities. Although FAP and DPPIV have dipeptidyl peptidase activity, the endopeptidase activity is specific to FAP, which is the basis for FAP detection and regulation (9, 13). Functional activation of FAP relies on its dimerization and glycosylation (14), and FAP can homodimerizes or heterodimerizes with DPPIV (15). In addition, FAP can bind to β-integrins, thereby enhancing the degradation and invasion ability of the extracellular matrix (16).

Under normal physiological conditions, FAP is expressed at low levels in most tissues. However, FAP expression is elevated across various tumor types in the breast, colorectal, pancreatic, lung, bladder, ovarian, and other organ cancers. In most epithelial carcinomas, FAP is primarily overexpressed in stromal cancer-associated fibroblasts (CAFs). FAP influences tumor growth through multiple mechanisms, including promoting proliferation, invasion, angiogenesis, epithelial-mesenchymal transition (EMT), stem cell promotion, immune suppression, and drug resistance (9). Therefore, in recent years, tumor assessment and treatments targeting FAP have been extensively studied and applied clinically, providing benefits to patients (17). In non-oncological diseases, FAP participates in multiple processes such as tissue remodeling, fibrosis, wound healing, inflammation, and atherosclerosis (9, 18–20). Furthermore, owing to its selective expression in repaired or reshaped tissues, FAP is considered a biomarker for activating fibroblasts in arthritis, autoimmune disorders, cardiovascular diseases (CVD), fibrosis, and metabolic diseases (21), since these nonmalignant diseases show an upgraded expression of FAP, which is associated with disease progression (20–23). For instance, the severity of liver fibrosis in patients with viral hepatitis C infection is correlated with the intrahepatic expression of FAP (21, 24). In a rat model of myocardial infarction (MI), cardiac FAP expression was significantly upregulated after MI, particularly in myofibroblasts in the area surrounding the infarction. In contrast, FAP-positive fibroblasts are present in the ischemic myocardial tissue of patients with MI, whereas FAP expression is not detected in healthy control heart specimens (25).

In 2006, Bauer et al. found that FAP was expressed in synovial samples from patients with RA and osteoarthritis (OA), with greater expression in patients with RA than in those with OA (6), confirming the association between FAP and arthritis. Moreover, inflammatory factors and chemokines secreted by activated FLS in RA promote and maintain joint inflammation (4, 5, 26–28). Consequently, previous studies have highlighted the important role of FAP in inflammation. In a mouse model of inflammatory arthritis lacking FAP, Wäldele et al. suggested that FAP deficiency improved joint inflammation and decreased cartilage destruction (29). Meanwhile, a clinical study on lung cancer has suggested that FAP is associated with an increase in the ratio of peripheral blood neutrophils to lymphocytes (30). Animal studies have also demonstrated that the depletion of FAP-positive FLS can attenuate the progression of arthritis (31). FAP has been identified as an osteogenesis-inhibiting molecule associated with bone destruction (32), with another study confirming its dual role in joint destruction and inflammatory processes (33). Thus, accumulating evidence has validated FAP as a critical biomarker of RA-FLS activation (19, 34). Therefore, as a surface marker of RA-FLS, FAP plays an important role in initiating abnormal immune responses and promoting RA development by mediating the interaction between RA-FLS and immune cells, regulating the differentiation, migration, and invasion of RA-FLS, facilitating the secretion of various cytokines, relieving contact inhibition, and inhibiting necrotic apoptosis (19, 34, 35). Since the introduction of the highly targeted small-molecule FAP inhibitor (FAPI) in 2010 (36), the number of studies on FAPI imaging has significantly increased. Studies have confirmed the value of targeted FAPI imaging in the field of arthritis, with elevated FAPI uptake being observed in joints of patients with OA (37, 38). Similarly, increased FAPI uptake has been observed in the sacroiliac and costovertebral joints in ankylosing spondylitis (AS), indicating sacroiliitis and spondylitis (39). Consequently, based on the high expression of FAP in RA-FLS, studies have explored the potential applications of FAPI in RA.

## Evolving role of targeted FAP imaging in the evaluation of RA

As mentioned above, the key effector cell of RA, FLS, highly express FAP (6–9), thus, researchers are now directing their focus on the application of FAP imaging in RA, especially in the evaluation of disease activity. Early research in this regard was primarily conducted using animal models. In 2015, Laverman et al. demonstrated the effectiveness of single-photon emission computed tomography (SPECT) and positron emission tomography (PET) in conjunction with the anti-FAP antibody ^111^In-28H1 for high-resolution visualization of arthritic joints in a collagen-induced arthritis (CIA) model. They also found that tracer accumulation correlated with the severity of joint inflammation (40). Similarly, Terry et al. found that ^111^In-28H1 uptake in joints correlated with the severity of arthritis, and etanercept treatment decreased the joint uptake of tracers in CIA mice (41). Consequently, studies have indicated that radiolabeled anti-FAP antibodies can serve as biomarkers to display disease activity and monitor treatment responses in arthritis, as seen in experimental arthritis models (42). In this regard, the development of FAP molecular probes and the pharmacokinetic properties of FAPI have facilitated further research on RA. In a pre-clinical study conducted by Ge et al. (43), an aluminum-[^18^F]-labeled 1,4,7-triazacyclononaneN,N′,N″-triacetic acid–conjugated FAP inhibitor 04 ([^18^F]AlF-NOTA-FAPI-04) was used to image RA-FLS in CIA mice and patients with RA. An *in vitro* experiment reported significantly increased binding of [^18^F]AlF-NOTA-FAPI-04 in RA-FLS. Compared to ^18^F-FDG, [^18^F]AlF-NOTA-FAPI-04 showed higher uptake in inflamed joints in the early stages of arthritis, which was positively correlated with arthritic scores. Additionally, [^18^F]AlF-NOTA FAPI-04 PET/CT imaging showed high tracer uptake in the synovium of the arthritic joints in two patients with RA. This suggests, that [^18^F]AlF-NOTA-FAPI-04 is a promising radiotracer for imaging RA-FLS and could potentially complement noninvasive diagnostic methods in RA.

In 2023, Luo et al. prospectively evaluated the efficacy of FAPI PET/CT for assessing joint disease activity in RA (44). This study compared the use of gallium-68 (^68^Ga)-FAPI with ^18^F-FDG PET/CT in 20 patients with RA, and demonstrated the potential of FAPI as a more precise imaging agent for RA. Although both PET/CT techniques can detect inflammation-affected joints, 15 of the 244 (6.1%) ^68^Ga-FAPI PET/CT-detected joints in six patients with RA were not displayed on ^18^F-FDG PET/CT, suggesting that ^68^Ga-FAPI PET/CT has a better sensitivity than ^18^F-FDG PET/CT. Additionally, ^68^Ga-FAPI uptake was higher than ^18^F-FDG for the affected joints in patients with RA. Moreover, the PET joint count and PET articular index of ^68^Ga-FAPI PET/CT correlated positively with disease activity and radiographic progression of joint damage. Another study also found that FAPI uptake in RA-FLS was positively correlated with the inflammatory phenotype and visually characterized the severity of arthritis (45). Furthermore, Mu et al. effectively identified joint involvement in patients with RA. The authors found that RA high disease activity was associated with increased standardized uptake value maximum (SUVmax), target-to-background ratio (TBR), [^18^F]FAPI–avid lesion volume (FLV), and total lesion FAP expression (TLF). Particularly, positive correlations were observed between a disease activity score of 28 using C-reactive protein (DAS28-CRP) and FLV and TLF, providing a non-invasive assessment of RA activity (46).

In addition to assessing disease activity, FAPI PET/CT also has the potential to diagnose RA. After injection of ^68^Ga-FAPI-04 CIA model, tracer uptake reached an increased level and retention was maintained in the swollen paws compared with healthy mice, revealing the diagnostic potential of ^68^Ga-FAPI-04 for detecting arthritis (47). A case report by Cheung et al. (48) demonstrated that ^68^Ga-FAPI PET/CT could be useful for detecting seronegative RA (SNRA). This is helpful in cases where patients pose negative for serum markers such as RF and ACPA, leading to significant challenges in diagnosis and management. Moreover, in clinical practice, patients with SNRA often experience delayed treatment, persistent disease activity, and eventual joint destruction due to missed and misdiagnoses (49, 50). These delays increase the likelihood of missed therapeutic opportunities and reduce the probability of achieving treatment targets (51). Therefore, the detection of arthritis via imaging is important for confirming the diagnosis. This case report used ^68^Ga-FAPI PET/CT in a 60-year-old female patient with SNRA and showed a symmetrically intense uptake in all the large joints, as well as the small joints of both hands, with a clear display of details in the joints (48).

Researchers have also explored the monitoring role of FAPI PET/CT in treatment response. Zhang et al. observed decreased signals in ^18^F-FAPI-04 PET imaging after four 4-week therapy with methotrexate or etanercept, suggesting that the detection of FAPI by PET imaging was more sensitive than the clinical arthritis score index in monitoring the response of RA to treatment (45). Accordingly, a recent prospective cohort study including 19 patients with RA investigated whether FAPI PET/CT could predict the treatment response. The results showed that, although responders and non-responders had similar clinical disease activity at baseline, responders had higher ^68^Ga-FAPI-04 uptake than non-responders (52).

The clinical applications of targeted FAP imaging in RA are listed in **Table 1**. However, two previous studies on FAPI PET/CT in patients with RA have reported instances of FAPI-positive uptake with asymptomatic clinical presentation (44, 52). This discrepancy in FAPI uptake may arise from several factors such as tenosynovitis, which commonly observed in patients with RA and may manifest as periarticular tenderness during physical examination (53). Moreover, inflammation in tendon sheaths near joints might fall outside the PET regions of interest (ROIs) delineated during imaging analysis. Additionally, clinically detected joint tenderness or swelling in some cases may stem from non-RA pathologies (e.g., OA) or may be confounded by comorbidities such as fibromyalgia. Consequently, these findings underscore the need for future studies to develop strategies for differentiating RA-related and non-RA joint pathologies using FAPI PET imaging and optimize ROI selection protocols to minimize diagnostic inaccuracies in RA activity assessment.

**Table 1:** List of clinical applications of targeted FAP imaging in rheumatoid arthritis.

Radiotracer	Articles	Possible diagnostic role	Assessment of disease activity	Potent therapeutic effect
^111^In-28H1	Laverman et al., 2015 (40)	+	+	
^111^In-28H1	Terry et al., 2016 (41)	+	+	
^99m^Tc-S-HYNIC-28H1	van der Geest et al., 2017 (42)		+	
[^18^F]AlF-NOTA-FAPI-04	Ge et al., 2022 (43)	+	+	
^68^Ga-FAPI	Luo et al., 2023 (44)	+	+	
^18^F-FAPI-04	Zhang et al., 2023 (45)	+	+	
^18^F-FAPI	Mu et al., 2025 (46)		+	
^68^Ga-FAPI-04	Tseng et al., 2025 (47)	+	+	
^68^Ga-FAPI	Cheung et al., 2023 (48)	+		
^68^Ga-FAPI-04	Pan et al., 2024 (52)		+	
FAP-ZF-NPs	Qi et al., 2023 (108)			+
28H1-IRDye700DX	Dorst et al., 2020 (109)			+
^68^Ga-FAPI-04/28H1-IRDye700DX	Dorst et al., 2022 (31)			+
^177^Lu-FAPI-04	Tseng et al., 2025 (47)			+

FAP, fibroblast activation protein; RA, rheumatoid arthritis; FAPI, fibroblast activation protein inhibitor; ^99m^Tc-S-HYNIC, ^99m^Tc-labeled succinimidyl-hydrazinonicotinamide; [^18^F]AlF-NOTA, aluminum-[^18^F]-labeled 1,4,7-triazacyclononaneN,N′,N″-triacetic acid; ZF-NPs, zinc ferrite nanoparticles.

## Targeted FAP imaging in RA related extra-articular manifestations

Patients with RA can have various extra-articular manifestations, especially those with insufficient treatment, including vasculitis, interstitial lung disease (ILD), amyloidosis, lymphoma, and CVD (1). ILD is the most common pulmonary complication of RA and the leading cause of mortality in patients with RA (54–56). ILD diagnosis requires a collaborative approach across multiple disciplines, including rheumatology, pulmonology, and radiology. Despite this, tools for early diagnosis and methods for prognostic prediction remain deficient. Although recent advancements, such as computer-aided tools and new imaging methods, are promising, they have not yet been widely adopted. For instance, high-resolution computer tomography (HRCT) is the gold standard imaging tool for ILD evaluation, however, it is limited by the use of radiation, which may not be conducive to the long-term follow-up of patients (especially pregnant patients) (57, 58). In contrast, chest radiography and pulmonary function have poor sensitivity for the diagnosis of ILD. While lung biopsy is an invasive procedure that can yield negative results. Lung ultrasound (LUS) depends on equipment and operator experience, which can affect the reliability of results (59, 60). Thus, a new quantitative tool is needed to supplement traditional visual assessments, offering the potential for improved diagnosis, monitoring, and management of ILD. Studies have reported that FAP is overexpressed in pulmonary fibrosis (61–63), particularly in regions of fibroblastic foci, where it plays a significant role in disease progression (64). Studies have demonstrated that FAPI tracer uptake via PET imaging is positive in fibrotic lung tissues (65, 66). Thus, FAPI PET/CT has been used to evaluate pulmonary manifestations in patients with connective tissues (65). In a single-center study of 21 patients with systemic sclerosis-associated ILD (SSc-ILD), the authors demonstrated that patients with SSc-ILD with higher disease activity had higher FAPI uptake, with the magnitude of tracer accumulation being correlated with the progression of ILD. Moreover, changes in FAPI uptake were concordant with the response to anti-fibrotic therapy, suggesting that FAPI imaging can assess fibrotic activity and treatment response (62). Besides being a noninvasive evaluation tool for fibrosis and inflammatory processes in patients with ILD, the findings of a recent prospective study conducted by Bahtouee et al. suggested that ^68^Ga-FAPI PET/CT may have the potential to differentiate levels of pulmonary fibrosis based on HRCT patterns (67). Röhrich et al. demonstrated that FAPI-PET imaging has the potential to differentiate between ILD and concurrent lung cancer using distinct signal patterns, as lung tumors are also visible on PET imaging (68).

Moreover, previous studies have shown that FAPI imaging can be used to detect myocardial injury of different etiologies because of the important role of activated fibroblasts in the repair and regeneration of damaged myocardium (69–71). RA significantly increases cardiovascular risks including heart failure, atherosclerosis, myocardial fibrosis, myocardial infarction, atrial fibrillation (72). Thus in 2023, Treutlein et al. revealed that FAPI uptake in the myocardial tissue of patients with SSc was positively correlated with cardiac disease activity, and that FAPI PET/CT exhibited greater sensitivity than cardiac magnetic resonance imaging for detecting cardiac injury (73). Furthermore, the study revealed a significant increase in FAPI uptake in patients with SSc and arrhythmia (73). Previous studies have also demonstrated that FAPI is linked to reparative fibrosis resulting from myocardial injury and reactive fibrosis induced by non-infectious inflammation (74). Consequently, these findings provide potential evidence for the use of FAPI-PET-CT for the evaluation of cardiac fibroblasts. Rheumatoid nodules are extraarticular manifestations of RA, which mimic neoplastic lesions in some, especially head and neck malignancy (75, 76). However, an increased uptake of the nodule was observed on ^18^F-FDG PET/CT in one case report (77). Thus, whether FAPI PET/CT can differentiate rheumatoid nodules from malignant lesions requires further investigation.

While the studies cited in this section primarily investigated FAPI imaging in non-RA pathologies such as SSc-ILD and cardiac fibrosis, their findings hold significant clinical relevance for understanding RA-associated complications. Patients with RA share pathophysiological mechanisms with conditions such as fibroblast activation and dysregulated tissue remodeling. Thus, the correlation between FAPI uptake and fibrotic activity in SSc-ILD provides a mechanistic framework for evaluating RA-ILD. Similarly, detection of myocardial fibroblast activation in SS using FAPI PET/CT can guide the early identification of RA-related cardiac fibrosis. Furthermore, Röhrich et al. highlighted the ability of FAPI to differentiate fibrotic lesions from malignancies, which is a critical consideration for patients with RA with rheumatoid nodules mimicking tumors. Although these studies did not directly involve RA cohorts, they established a proof-of-concept for the utility of FAPI in mapping fibroblast-driven pathology across diseases. Consequently, future research should validate these approaches in RA-specific populations to refine the diagnostic and prognostic strategies for RA complications, leveraging insights from analogous autoimmune and fibrotic disorders.

## Targeted FAP therapy for RA

Despite advances in RA management, significant limitations remain. For instance, conventional therapies, such as nonsteroidal anti-inflammatory drugs (NSAIDs) and conventional synthetic disease-modifying antirheumatic drugs (csDMARDs), are often hampered by prolonged drug onset and adverse effects (e.g., gastrointestinal reactions, hepatotoxicity, and myelosuppression), making them ineffective in achieving remission in refractory cases. Biologic DMARDs (bDMARDs), including tumor necrosis factor (TNF)-α antagonists and interleukin (IL)-6 inhibitors, and targeted synthetic DMARDs (tsDMARDs) including Janus kinase(JAK) inhibitors rapidly suppress acute inflammation and improve outcomes. However, the use of bDMARDs and tsDMARDs in the treatment of chronic inflammatory diseases is limited by drug resistance and substantial safety risks including infection, thromboembolic events, cardiovascular toxicity, and malignancies (78, 79). Additionally, injecting glucocorticoids into the joint cavity or performing synovectomy only provides short-term relief from symptoms and does not inhibit the continuous progression of synovial vascular opacities, which cannot prevent further joint damage. Thus, more specific and precise therapeutic targets are required for targeted therapy, along with identification of surface biomarkers of activated RA-FLS.

As a novel targeted molecular probe, FAP imaging has confirmed the high uptake of FAP by tumors and its low expression in healthy tissues, making it an excellent target for targeted radionuclide therapy (TRT), a therapeutic approach that utilizes specific small molecules or peptides to bind to receptors or biomarkers on diseased cells, enabling the localized accumulation of radionuclides around pathological sites. The radiation energy emitted during radionuclide decay is then harnessed to destroy the targeted diseased cells, thereby achieving therapeutic efficacy (80–83). TRT has been successfully applied in oncology, with notable examples. ^177^Lu-DOTATATE was the first approved radiopharmaceutical, targeting somatostatin receptors (SSTRs), used for treating gastroenteropancreatic neuroendocrine tumors (80). Similarly, ^177^Lu-PSMA-617, a therapeutic agent targeting prostate-specific membrane antigens (PSMA), has been approved for treating recurrent or refractory metastatic prostate cancer (81). FAP-targeted radionuclide therapy (FAP-TRT) involves the conjugation of small molecule inhibitors or peptides that specifically bind FAP with therapeutic radionuclides to construct radiopharmaceutical agents. This strategy enables the precise delivery of radiation to FAP-expressing targets, achieving localized therapeutic effects while minimizing systemic toxicity associated with whole-body radiation exposure (84, 85). The targeted therapeutic effect of FAP is achieved through the following mechanisms: enzyme activity inhibition, FAP protease activity blocking via small molecules or antibodies; prodrug activation, utilization of FAP protease properties to cleave antitumor prodrugs coupled with their targeted peptides; immune regulation-developing vaccine therapy for FAP; and cell therapy-FAP-targeted chimeric antigen receptor (CAR)-T cell therapy (9, 21). In terms of enzyme activity inhibition, talabostat is one of the first small molecules to inhibit the dipeptidyl peptidase activity shared by DPPIV and FAP, showing a good anti-tumor effect (86–88). Sibrotuzumab, a humanized monoclonal anti-FAP antibody, enhanced cytotoxic activity against FAP-expressing tumor cells in *in vitro* experiments, but had no therapeutic effect observed in clinical studies (89–92). Prodrugs conjugated to FAP substrates significantly reduced tumor growth and drug toxicity to organs (93–95). Similarly, FAP vaccination decreased tumor growth, suppressed metastasis, and increased survival (96–99). CAR T-cell therapy has already been approved for the treatment of some forms of leukemia and lymphoma (100). Schuberth et al. first used FAP CAR-T cell therapy to eliminate FAP^+^ tumor cells in pleural mesothelioma (MPM) and achieved therapeutic effects *in vivo* and *in vitro* (101). Subsequent studies also found that FAP CAR-T cells can inhibit tumor growth and improve outcomes (102, 103). Furthermore, Aghajanian et al. demonstrated that FAP CAR-T cells can reduce cardiac fibrosis (104), expanding the application of targeted FAP therapy.

**Figure 1:**
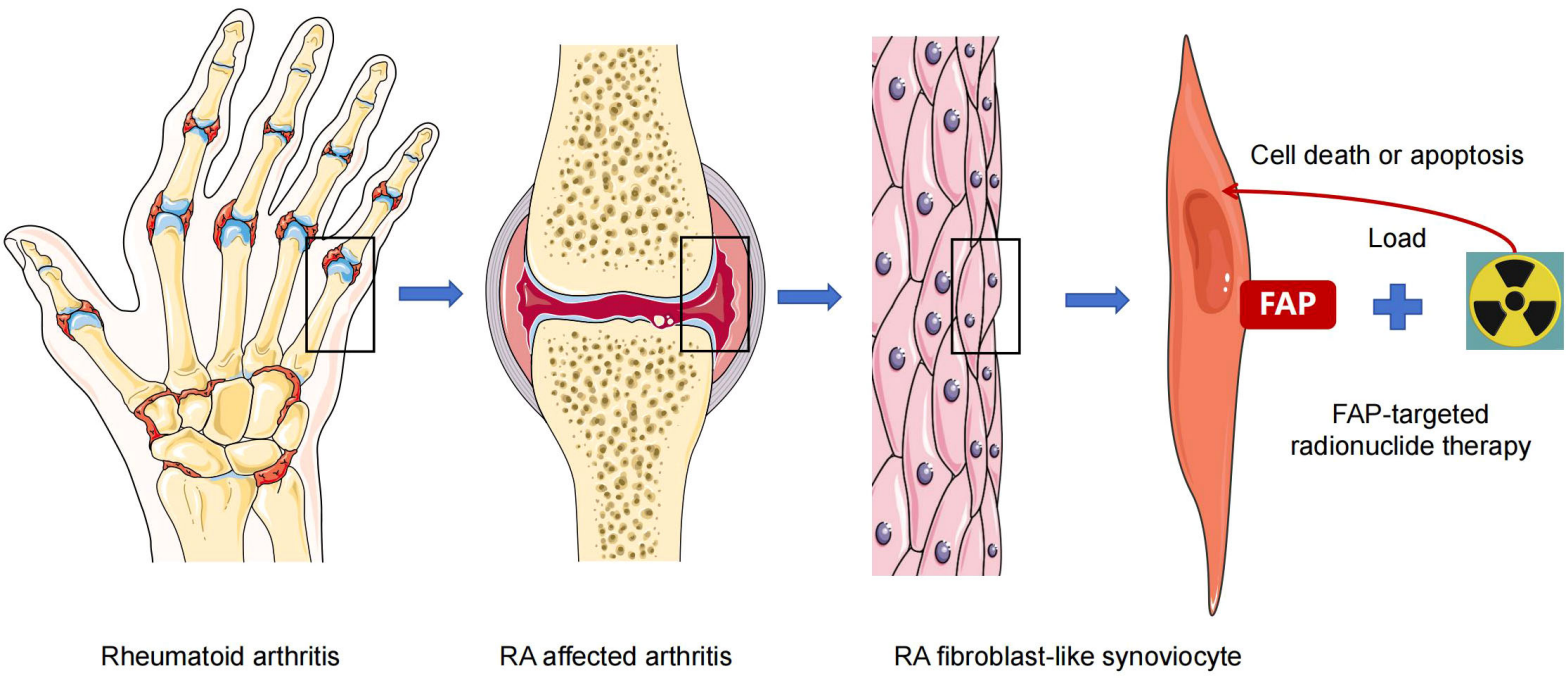
Therapeutic effect of FAP-targeted radionuclide therapy for RA.

As stated earlier, FAP is overexpressed in RA-FLS (6–9, 21) and is associated with the invasive phenotype of FLS, which aggravates cartilage degradation (105–107). Additionally, animal models and human imaging trials have confirmed that FAP can precisely detect RA-FLS (43, 44). Therefore, similar to targeted removal of FAP positive tumor cells, targeted clearance of RA-FLS may become a promising option for treating RA (**Figure 1**). Previous studies have shown that the deletion of FAP^+^ fibroblasts ameliorated inflammation, reduced bone erosion, and improved tissue damage in mice (29, 33). In animal experiment, FAP-targeted zinc ferrite nanoparticles (ZF-NPs) significantly improved synovitis, reduced angiogenesis in synovial tissue, suppressed articular cartilage damage, and inhibited macrophage infiltration in mice with adjuvant-induced arthritis (108), demonstrating the potential utility of FAP-ZF-NPs in the treatment of RA. In this study, ZF-NPs and FAP peptide were engineered to target FAP positive FLS. The complex enhanced RA-FLS apoptosis by activating the endoplasmic reticulum stress (ERS) system and promoting mitochondrial damage, thereby inhibiting the process of synovitis. Dorst et al. evaluated a therapeutic approach in RA guided by FAPI PET/CT, which was advantageous over traditional imaging techniques. In 2020, the researchers demonstrated that FAP-targeted photodynamic therapy (FAP-tPDT) could selectively eliminate FAP-positive fibroblasts *in vitro* using a 3T3 cell line stably transfected with FAP, and *in vivo* using a CIA model (109). The authors used anti-FAP antibody 28H1 conjugated with the photosensitizer IRDye700DX to successfully induce FAP-specific cell death and delayed arthritis. Later, based on the above study, the authors used FAP-tPDT for clinical exploration that was closer to patients with RA and performed *in vitro* experiments on synovial fibroblasts derived from the synovial tissues of three patients with RA. They showed that after incubation with the 28H1-IRDye700DX, FAP-positive fibroblasts exhibited a gradual decrease in cell viability. Furthermore, upregulated markers of cell damage and death were observed in RA synovial tissues after FAP-tPDT, with no adverse effects in the macrophages of neighboring synovial fibroblasts (31). Hence, the authors concluded that FAP-tPDT selectively increased cytotoxicity and induced cell death in human RA synovial fibroblasts without any systemic side effects. Targeted radiotherapy can destruct cellular DNA and generate free radicals through radioactive ionization, resulting in cell death. Thus, via removing pro-inflammatory cells or tissues, targeted radiotherapy may achieve an anti-inflammatory effect in arthritis. Previously, Weissmann et al. reported that low-dose radiotherapy leaded a systemic anti-inflammatory effect and reduced pain in OA (110). More recently, Tseng et al. explored the therapeutic potential of ^177^Lu-labelled FAPI-04 in CIA and found that ^177^Lu-FAPI-04 therapy reduced the arthritis scores in mice. Further analysis revealed that mice injected with ^177^Lu-FAPI-04 showed a significant decrease in thymocyte differentiation antigen-1 (Thy-1) positive FAP^+^ FLS cells, Th1 cells and Th17 cells as well as TNF-α and IL-1β in serum (47).

Preclinical studies, including those using FAP-tPDT, have shown a significant reduction in synovitis and angiogenesis in RA models. Additionally, early phase clinical trials further support the feasibility of selectively ablating pathogenic RA-FLS with minimal off-target effects. Moreover, recent advancements in FAP-targeted therapies for RA have yielded promising results. However, the current research predominantly relies on animal models and exploratory clinical trials, and large-scale human studies are limited. The presence of confounding factors such as heterogeneity in patient populations and comorbidities may compromise the validity of the conclusions. Therefore, future research can be explored from the following perspectives: combining nanotechnology to develop FAP-targeted drug delivery systems to improve treatment accuracy and integrating single-cell sequencing technology to analyze the heterogeneity of FAP^+^cell sub-populations to guide personalized treatment. Nevertheless, further validation through large-scale prospective studies is imperative to confirm the therapeutic efficacy and translate preclinical success into routine clinical practice.

## Conclusion and prospective

The FAP-targeted imaging technology has revived the realms of molecular and precision imaging. FAPI, a technology that has been successfully applied in tumor diagnosis and treatment, shows promise for visualizing myocardial fibrosis, and has potential applications in autoimmune diseases and other non-tumor conditions. However, current research efforts are hampered by limited sample sizes, retrospective study designs, and inadequate histopathological validation. Additionally, the dual expression of FAP in benign and malignant lesions requires refined imaging protocols to increase its specificity. Therefore, future studies should clarify the interplay between FAP and inflammatory signaling pathways to discover synergistic therapeutic targets. Moreover, comparisons of FAPI variants and antibody-based probes are required to optimize imaging protocols. Large-scale clinical trials are essential to validate the prognostic significance of FAP expression levels compared to traditional biomarkers such as ACPA, and to incorporate artificial intelligence-driven tools for PET/CT analysis, which are used to evaluate RA. Furthermore, addressing safety concerns, particularly the long-term effects of radionuclide therapy, requires extended follow-up and multidisciplinary collaboration between radiobiologists, bioengineers, and rheumatologists.
